# Supplementary material for: In vitro expansion of pancreatic islet clusters facilitated by hormones and chemicals
Source: Cell Discov. 2020 Apr 7;6:20. doi: 10.1038/s41421-020-0159-x (PMC7136205; doi:10.1038/s41421-020-0159-x)
Supplement: Supplementary file 1 — Supplementary Materials for In vitro pancreatic islet cluster expansion facilitated by hormones and chemicals [file 41421_2020_159_MOESM1_ESM.pdf]

Supplementary Information

Supplementary Fig 1

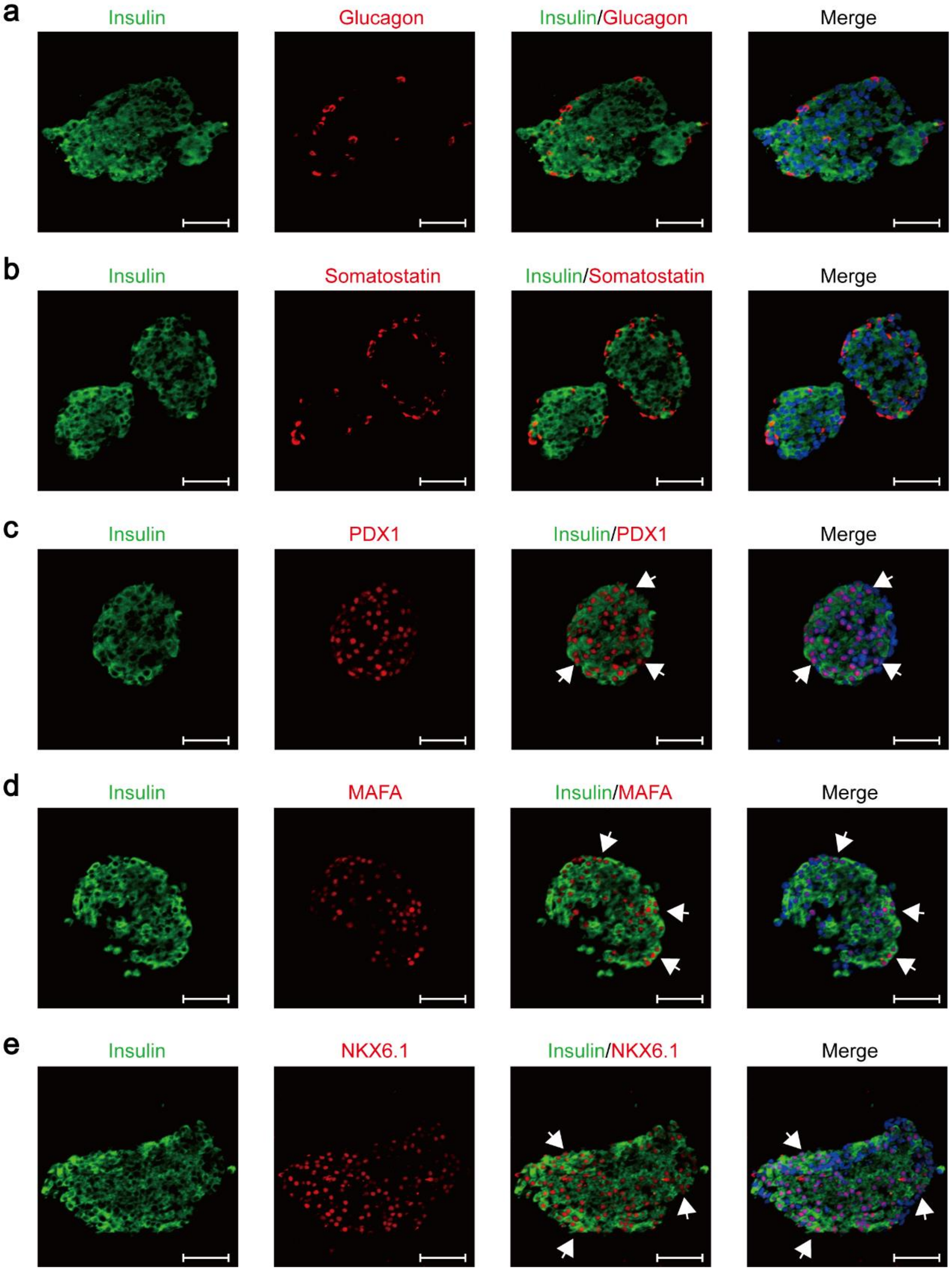

Supplementary Fig. S1: Immunostaining for pancreatic islet cell markers of in vitro primary islets. Related to Fig. 1.

a Immunofluorescence staining for insulin and glucagon in primary islets derived from pregnant mice; the scale bar represents 50 μm.

b Immunofluorescence staining for insulin and somatostatin in primary islets derived from pregnant mice; the scale bar represents 50 μm.

c Immunofluorescence staining for insulin and PDX1 in primary islets derived from pregnant mice; the scale bar represents 50 μm.

d Immunofluorescence staining for insulin and MAFA in primary islets derived from pregnant mice; the scale bar represents 50 μm.

e Immunofluorescence staining for insulin and NKX6.1 in primary islets derived from pregnant mice; the scale bar represents 50 μm.

Supplementary Fig 2

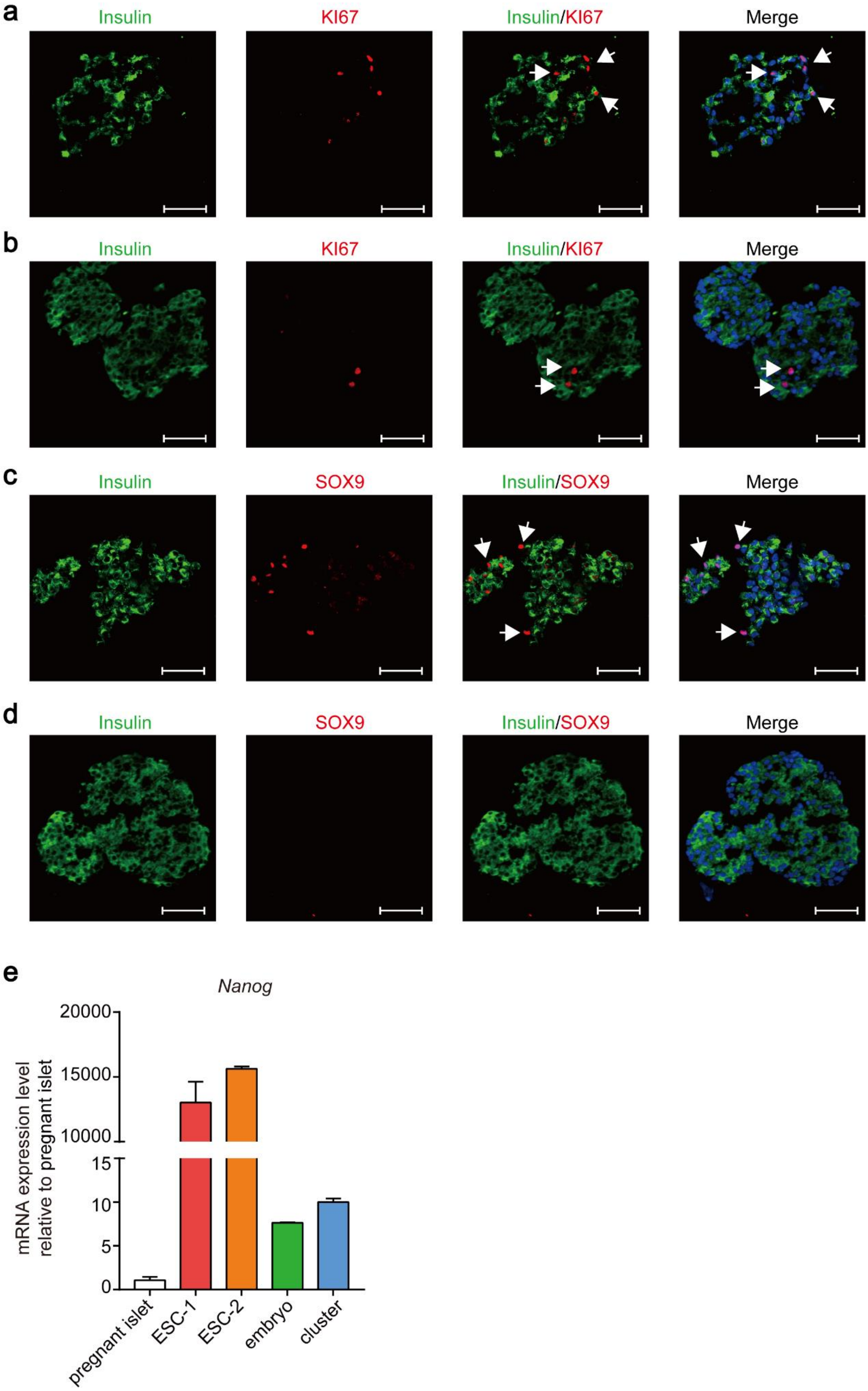

Supplementary Fig. S2: Further analysis of gene expression in expanded clusters compared to primary islets. Related to Fig. 5.

a Immunofluorescence staining for KI67 in expanded pancreatic islet clusters derived from pregnant mice; the scale bar represents 50 μm.

b Immunofluorescence staining for KI67 in primary islets derived from pregnant mice; the scale bar represents 50 μm.

c Immunofluorescence staining for SOX9 in expanded pancreatic islet clusters derived from pregnant mice; the scale bar represents 50 μm.

d Immunofluorescence staining for SOX9 in expanded pancreatic islet clusters derived from pregnant mice; the scale bar represents 50 μm.

e Comparison of the *Nanog* expression. qRT-PCR analysis of the Nanog expression in different cells and tissues. ESC-1 and ESC-2: two populations of embryonic stem cells of C57BL/6 mice; embryo: E11.5 embryo of C57BL/6 mice; cluster: day 7 expanded islet clusters derived from pregnant mice

Supplementary Tables

Supplementary Table S1. Primer sequences used in this study

Primers	Sequences
Gcg Forward for mouse cDNA:	GGCACATTCACCAGCGACTA
Gcg Reverse for mouse cDNA:	GAGAAGGAGCCATCAGCGTG
Ins1 Forward for mouse cDNA:	TGGACTATAAAGCTGGTGGGC
Ins1 Reverse for mouse cDNA:	TGTGTAGAAGAAGCCACGCT
Ins2 Forward for mouse cDNA:	GCTATCCTCAACCCAGCCTAT
Ins2 Reverse for mouse cDNA:	CTCCAGTGCCAAGGTCTGAA
Mafa Forward for mouse cDNA:	GGCACATTCTGGAGAGCGAG
Mafa Reverse for mouse cDNA:	TCACAGAAAGAAGTCGGGTGC
Mafb Forward for mouse cDNA:	TACCAGCAGATGAACCCCGA
Mafb Reverse for mouse cDNA:	GCTAGTGGGTAGCTGTTGCG
Sst Forward for mouse cDNA:	CCACCGGGAAACAGGAACTG
Sst Reverse for mouse cDNA:	TTGCTGGGTTCGAGTTGGC
Ngn3 Forward for mouse cDNA:	ACGCAATTTACTCCAGGCGA
Ngn3 Reverse for mouse cDNA:	GAGGCGCCATCCTAGTTCTC
Hlxb9 Forward for mouse cDNA:	GAACCTCTTGGGGAAGTGCC
Hlxb9 Reverse for mouse cDNA:	TCTTTGGCCTTTTTGCTGCG
Gata4 Forward for mouse cDNA:	AGATCGCGCCGGTTTTCTG
Gata4 Reverse for mouse cDNA:	GATCACCCACCGGCTAAAGA
Gata6 Forward for mouse cDNA:	TTTCCGGCAGAGCAGTAAGAG
Gata6 Reverse for mouse cDNA:	GAAACGCTTTGGCAGGCAC
Sox9 Forward for mouse cDNA:	CGGAACAGACTCACATCTCTCC
Sox9 Reverse for mouse cDNA:	GCTTGCACGTCGGTTTTGG
Ki67 Forward for mouse cDNA:	CAAGGCGAGCCTCAAGAGATA
Ki67 Reverse for mouse cDNA:	TGTGCTGTTCTACATGCCCTG
Nanog Forward for mouse cDNA:	AGGACAGGTTTCAGAAGCAGA
Nanog Reverse for mouse cDNA:	CCATTGCTAGTCTTCAACCACTG

Ccnb1 Forward for mouse cDNA:	GCGTGTGCCTGTGACAGTTA
Ccnb1 Reverse for mouse cDNA:	CCTAGCGTTTTTGCTTCCCTT
Ccnd1 Forward for mouse cDNA:	TGACTGCCGAGAAGTTGTGC
Ccnd1 Reverse for mouse cDNA:	CTCATCCGCCTCTGGCATT
Pcna Forward for mouse cDNA:	TTGCACGTATATGCCGAGACC
Pcna Reverse for mouse cDNA:	GGTGAACAGGCTCATTCATCTCT
Pdx1 Forward for mouse cDNA:	CCTTTCCCGAATGGAACCGA
Pdx1 Reverse for mouse cDNA:	GGGCCGGGAGATGTATTTGT
Sox17 Forward for mouse cDNA:	CCAAAGCGGAGTCTCGCAT
Sox17 Reverse for mouse cDNA:	GCCTAGCATCTTGCTTAGCTC
Cdk4 Forward for mouse cDNA:	GCCACTCGATATGAACCCGT
Cdk4 Reverse for mouse cDNA:	CACAGACATCCATCAGCCGT
Gcg Forward for rat cDNA:	TTCACAGGGCACATTCACCA
Gcg Reverse for rat cDNA:	CTATGGCGACTTCTTCCGGG
Ins1 Forward for rat cDNA:	ACCCTAAGTGACCAGCTACAATC
Ins1 Reverse for rat cDNA:	CGGGTCCTCCACTTCACGAC
Ins2 Forward for rat cDNA:	ACCTTTGTGGTTCTCACTTGGT
Ins2 Reverse for rat cDNA:	CAGTGCCAAGGTCTGAAGGTCA
Mafa Forward for rat cDNA:	GACAAGTTTGCGCAGGCCG
Mafa Reverse for rat cDNA:	GTATTCACCGTTCTCGGGGC
Mafb Forward for rat cDNA:	GCAACGGTAGTGTGGAGGAC
Mafb Reverse for rat cDNA:	GAGCTGCGTCTTCTCGTTCT
Sst Forward for rat cDNA:	GCTACTGGAGTCGTCTCTGC
Sst Reverse for rat cDNA:	GGCATCGTTCTCTGTCTGGT
Ngn3 Forward for rat cDNA:	GTTCCAATTCCACCCCACCT
Ngn3 Reverse for rat cDNA:	CGCAGGGTCTCGATCTTTGT
Hlxb9 Forward for rat cDNA:	CGGCGCTTTCCTACTCGTAT
Hlxb9 Reverse for rat cDNA:	TCCCCAAGAGGTTCGATTGC
Gata4 Forward for rat cDNA:	TGAATGGTATCAACCGGCCC

Gata4 Reverse for rat cDNA:	TTTGAATCCCCTCCTTCCGC
Gata6 Forward for rat cDNA:	TCATCACCACCCGACCTACT
Gata6 Reverse for rat cDNA:	GCATGCGTTGCACAGGTAAT
Sox9 Forward for rat cDNA:	CACAAGAAAGACCACCCCGA
Sox9 Reverse for rat cDNA:	TGCACGTCTGTTTTGGGAGT
Ki67 Forward for rat cDNA:	ACAGGGCTTAGGAAACAGTCC
Ki67 Reverse for rat cDNA:	GGTTCTAACTGGTCTTCCTGGT
Nanog Forward for rat cDNA:	AAGTCCCTTCCCTTGCCGT
Nanog Reverse for rat cDNA:	CTCGGGACCAGACAGCTTTAG
Ccnb1 Forward for rat cDNA:	GGGTGTCTTCTCAGATCGGC
Ccnb1 Reverse for rat cDNA:	TCCACAGGTTTTGGTAGGGC
Ccnd1 Forward for rat cDNA:	TCAAGTGTGACCCGGACTG
Ccnd1 Reverse for rat cDNA:	CTACTTGGTGACTCCCGCCT
Pcna Forward for rat cDNA:	GACGGGGTGAAGTTTTCTGC
Pcna Reverse for rat cDNA:	GACAGTGGAGTGGCTTTTGTG

Supplementary Table S2. Antibodies used in this study

Antibodies	Source	Identifier
Anti-insulin	Santa	sc-9168
Anti-glucagon	Sigma	G2654
Anti-somatostatin	Abcom	ab30788
Anti-PDX1	Abcom	ab47267
Anti-SOX9	Abcom	ab185966
Anti-NKX6.1	Abcom	ab221549
Anti-MAFA	Abcom	Ab26405
Anti-KI67	Cell Signaling Technology	D3B5
Donkey anti-Mouse IgG (H+L) (secondary antibody)	Invitrogen	A10037
Donkey anti- Rabbit IgG (H+L) (secondary antibody)	Invitrogen	A21206
Donkey anti-Rat IgG(H + L) (secondary antibody)	Abcam	ab150154

Supplementary Table S3. Chemicals, Peptides, and Recombinant Proteins used in this study

Reagents	Source	Identifier
TRIzol	Thermo	15596-026
Cell Recovery Solution	Corning	354253
Matrigel	Corning	356231
GlutaMax	Thermo	35050-061
Penicillin-Streptomycin	Biological Industries	03-031-1B
recombinant human EGF	Peprotech	AF-100-15
recombinant human FGF10	Peprotech	100-26
CHIR-99021	BioGems	2520691-1MG
N-Acetylcysteine	Sigma-Aldrich	A9165-25G
Nicotinamide	Sigma-Aldrich	N0636
B27 Supplement (minus Vitamin A)	Thermo	12587-010
gastrin-1, human	MedChemExpress	HY-P1097
A83-01	Adooq Bioscience	A12358
Y-27632	Adooq Bioscience	A11001
Forskolin	TargetMol	T2939
Exendin4	ChinaPeptides	
5-Iodotubercidin	Adooq Bioscience	A13948
L-Ascorbic acid	Sigma-Aldrich	A4544
Triton X-100	Solarbio	T8200
Donkey Serum	Solarbio	SL050
DAPI	Beyotime	C1002
